# Phenotypic and Whole-Genome Sequencing-Based Profiling of Antimicrobial Resistance and Virulence in *Pseudomonas aeruginosa* Isolated from Patients with Ventilator-Associated Pneumonia and Ventilator-Associated Tracheobronchitis in a Croatian Intensive Care Unit

**DOI:** 10.3390/genes17020130

**Published:** 2026-01-26

**Authors:** Marija Cavka, Marija Kvesic Ivankovic, Ana Maravic, Mia Dzelalija, Jelena Marinovic, Ivana Goic-Barisic, Marija Tonkic, Anita Novak

**Affiliations:** 1 Department of Anesthesiology and Intensive Care, University Hospital of Split, Spinciceva 1, 21 000 Split, Croatia; 2 Institute of Oceanography and Fisheries, Setaliste Ivana Mestrovica 63, 21 000 Split, Croatia; 3 Department of Biology, Faculty of Science, University of Split, Rudera Boskovica 33, 21 000 Split, Croatia; 4 Department of Clinical Microbiology, University Hospital of Split, Spinciceva 1, 21 000 Split, Croatia; 5 School of Medicine, University of Split, Soltanska 2A, 21 000 Split, Croatia

**Keywords:** *Pseudomonas aeruginosa*, multidrug-resistance, hospital-acquired pneumonia, intensive care unit, whole-genome sequencing, VIM-2, genetic context

## Abstract

Background/Objectives: *Pseudomonas aeruginosa* is one of the leading causes of ventilator-associated pneumonia (VAP) and ventilator-associated tracheobronchitis (VAT), with a worldwide spread of difficult-to-treat high-risk clones. This study aimed to investigate the virulence potential and to characterize phenotypic and genotypic antimicrobial resistance (AMR) in *P. aeruginosa* causing VAP/VAT in the Intensive Care Unit (ICU), University Hospital of Split, Croatia. Methods: The study included *P. aeruginosa* isolates obtained from ICU patients who met the criteria for VAP or VAT, between January 2023 and January 2024. Isolates were identified using MALDI-TOF MS and tested for antimicrobial susceptibility (AST). A subset of phenotypically multidrug-resistant (MDR) isolates was further analyzed using whole-genome sequencing (WGS) and multilocus sequence typing. Results: A high rate of resistance was detected to ceftazidime (23.4%), imipenem (39.6%), and meropenem (43.8%). WGS confirmed the presence of multiple AMR genes, including the *bla*_VIM-2_ gene, whose genetic environment highlights a complex MDR locus integrating multiple AMR determinants and mobile genetic elements. All tested isolates possessed genes for class C (*bla*_PDC34_, *bla*_PDC374_ or *bla*_PDC16_) and class D (*bla*_OXA-2_, *bla*_OXA-10_ or *bla*_OXA-50_) β-lactamases, *fosA*, *aph(3′)-IIb* and *crpP* genes. Additionally, WGS analysis revealed the presence of numerous virulence genes including those for adherence (Type IV pili and Fap protein production), motility (such as *flgF*), biofilm formation (e.g., *algE* and *mucE*), quorum sensing (*lasI*, *lasR*, *rhlI* and *rhlR*), exotoxin (*toxA* and *plcH*) and exoenzyme activity (*exoU*, *exoT*, *exoS*, *exoY*, *pcrV*, *hcp1* and *lasA*). The isolates belonged to four different sequence types: ST235, ST446, the high-risk ST253 and the widely distributed ST395. Phylogenomic comparison demonstrated that the isolates from this study do not originate from a single clonal source, but instead represent multiple globally distributed high-risk *P. aeruginosa* lineages introduced into the clinical setting. Conclusions: Due to the emergence of high-risk clones with broad AMR and strong virulence potential, ineffectiveness of standard empirical therapy may be anticipated, highlighting the urgent need for new therapeutic approaches (including those targeting major virulence factors).

## 1. Introduction

*Pseudomonas aeruginosa* (*P. aeruginosa*) is a Gram-negative, non-fermenting bacillus, that can often be found in moist environments, soil and on plants. It is considered a common colonizer and an opportunistic pathogen in humans [1,2]. Numerous virulence factors, particularly the capacity for biofilm formation, as well as antimicrobial resistance (AMR), make this opportunistic microorganism one of the most important causes of hospital-acquired infections (HAI) with high morbidity and mortality rates [3].

There are many mechanisms, ranging from efflux pumps to the production of β-lactamases and gene mutations, that result in resistance not only to cephalosporins, which were traditionally the first-line therapy, but also to a broad range of antibiotics, posing a significant challenge for treatment [3,4,5]. Overproduction of its naturally occurring AmpC-type chromosomally-encoded cephalosporinase (so-called *Pseudomonas*-derived cephalosporinase, PDC) is one of the major mechanisms responsible for broad AMR (piperacillin, ceftazidime, cefepime, aztreonam) and even leads to reduced carbapenem susceptibility [6]. However, resistance to carbapenems is mainly linked to the production of metallo-β-lactamase (MBL) carbapenemases [7]. Carbapenem-resistant *P. aeruginosa* is classified by the World Health Organization (WHO) as a difficult-to-treat (DTT), high-risk priority pathogen of global concern, requiring the development of new antimicrobials [3,8,9].

Genome plasticity makes this pathogen metabolically versatile, with a high capacity for adaptation to different conditions and environments. For instance, cumulative mutations may increase the likelihood of the emergence of new bacterial variants with different expression of virulence genes and the antimicrobial resistome [10]. The intensive care unit (ICU) is a high-risk environment for infections caused by multidrug-resistant (MDR) microorganisms, such as *P. aeruginosa* [11]. Underlying comorbidities, immunosuppression, and the use of various medical devices (especially catheters and mechanical ventilation) are additional risk factors for the development of severe, life-threatening ICU infections [4].

*P. aeruginosa* is one of the leading causes of ventilator-associated pneumonia (VAP) and ventilator-associated tracheobronchitis (VAT), which are two types of HAI that occur in patients within the ICU [12,13]. Although VAT and VAP can occur independently of each other, substantial evidence supports the notion that VAT is likely to progress to pneumonia if left untreated or improperly managed [14]. Mortality rates associated with VAP range widely between 24% and 76%, as they are closely linked to the severity of the underlying illness and the diagnostic variability among ICU patients. VAP significantly contributes to antibiotic prescribing in the ICU, where the extensive use of antimicrobial drugs drives the rising incidence of infections caused by MDR pathogens, leading to further increases in morbidity and mortality rates [15,16].

Of particular concern is the emergence of high-risk *P. aeruginosa* clones and their worldwide distribution, which represents a major challenge for global healthcare society [10]. Understanding the genetic variability among different strains of *P. aeruginosa* is crucial for gaining deeper insights into their local and global distribution and transmission. Recently, whole-genome sequencing (WGS) and bioinformatics have become increasingly prevalent for these analyses [3]. Genotyping can facilitate more precise antimicrobial treatment and prevent colonization and consequent severe infections with fatal outcomes.

Although VAP is one of the most common HAIs in the ICU, there are limited data on etiology and antimicrobial susceptibility from the University Hospital of Split, Croatia, without in-depth molecular analysis [17]. The present study aimed to characterise phenotypic and genotypic AMR in *P. aeruginosa* causing VAP/VAT in the ICU, University Hospital of Split, Croatia, as well as to explore virulence potential using WGS, emphasizing the high MDR and virulence potential of this ubiquitous microorganism.

## 2. Materials and Methods

The inclusion criteria were diagnosed nosocomial lower respiratory tract infection (LRTI), including ventilator-associated pneumonia (VAP) or ventilator-associated tracheobronchitis (VAT), in ICU patients and isolation of *P. aeruginosa* from bronchoalveolar lavage (significant threshold ≥ 10^4^ colony-forming units (CFU)/mL) or tracheal aspirate (significant threshold ≥ 10^6^ CFU/mL) from January 2023 to January 2024.

LRTIs were diagnosed by ICU clinicians according to clinical, laboratory and radiological findings and classified as follows:

• Definitions of nosocomial lower respiratory tract infections (LRTIs)

Ventilator-associated pneumonia (VAP) is an LRTI developed in patients admitted to the ICU ≥ 48 h of tracheal intubation or tracheostomy with a radiological pulmonary infiltrate.

Ventilator-associated tracheobronchitis (VAT) is an LRTI developed in patients admitted to the ICU ≥ 48 h of tracheal intubation or tracheostomy without a new or progressive radiological pulmonary infiltrate [18,19,20].

• Cultivation and identification

Bronchial and tracheal aspirates were quantitatively inoculated onto blood agar (BA) in 10% CO_2_ and nonselective CHROMagar Orientation (CHROMagar, Paris, France) medium under aerobic conditions at 37 °C for 24 h and then reincubated for another 24 h under the same conditions. Isolates were identified using matrix-assisted laser desorption ionization mass spectrometry (MALDI-TOF MS, MALDI Biotyper^®^ sirius, Bruker, Bremen, Germany). Antimicrobial susceptibility testing (AST) was performed for antipseudomonal cephalosporins and penicillins (piperacillin/tazobactam (P/T), ceftazidime (CAZ), cefepime (CEF), ceftazidime/avibactam (CAZ/A), ceftolozane/tazobactam (C/T) and cefiderocol (FDC), quinolones (ciprofloxacin, CIP, and levofloxacin, LEV), carbapenems (imipenem (IMI) and meropenem, MEM) and aminoglycosides (amikacin (AMK) and tobramycin, TBR), using the disc diffusion method (Liofilchem, Roseto degli Abruzzi, Italy) and interpreted as susceptible (S), susceptible with increased exposure (I) or resistant (R), according to EUCAST (v. 13.0) [21]. Additionally, broth microdilution (BMD) for susceptibility testing of colistin was performed on a subset of multidrug-resistant isolates according to EUCAST guidelines [22]. Multidrug resistance (MDR) was defined as acquired non-susceptibility to at least one agent in three or more antimicrobial categories [23].

• Genomic DNA Extraction and Whole-Genome Sequencing

According to the phenotypic resistance profiles, genomic DNA from five *P. aeruginosa* MDR isolates (VAP-PA-1–VAP-PA-5) was extracted using the NucleoSpin Microbial DNA Kit (Macherey-Nagel, Cambridge, UK). The concentration and quality of the extracted DNA were assessed using a NanoDrop^®^ 1000 Spectrophotometer (Thermo Scientific, Waltham, MA, USA). Genomic DNA was sent to Novogene (Cambridge, UK) for WGS. DNA libraries were prepared using the NEBNext^®^ DNA Library Prep Kit (Illumina, San Diego, CA, USA) according to the manufacturer’s instructions. After library quantification by Qubit fluorometer and qPCR, paired-end sequencing (2 × 150 bp) was performed on the Illumina NovaSeq 6000 platform. Raw reads were subjected to quality control to remove adapter contamination, reads containing more than 10% ambiguous nucleotides (N), and reads with more than 50% of bases having a Phred score ≤ 5.

• Genome Analysis

De novo genome assemblies were generated from paired-end reads using Shovill v0.9.0 with subsampling of read depth down to 150× and assembly using SPAdes v.4.0. The resulting contigs were annotated with Prokka (v1.14.6). Functional genomic characterization was performed using the ABRicate tool (v1.0.1). Assembled genomes were screened against ABRicate databases, including AMRFinderPlus, CARD and VFDB, to identify antimicrobial resistance genes (ARGs), virulence factors, and other relevant genetic elements [24,25,26].

Genomic similarity between the five VAP-associated *P. aeruginosa* isolates and 1225 publicly available complete *P. aeruginosa* RefSeq genomes was assessed using MASH v2.3 and average nucleotide identity (ANI) calculated with fastANI.

Multilocus sequence typing (MLST) was determined using genomic sequences as input data against the standard MLST schemes [25].

A core gene alignment of the five *P. aeruginosa* isolates was generated using Roary v3.13.0 with the MAFFT alignment algorithm. A maximum-likelihood phylogenetic tree was then constructed in IQ-TREE v2.3.3, using ModelFinder to select the best-fit substitution model and performing 1000 ultrafast bootstrap replicates. The resulting tree, annotated with sequence type (ST) profiles, was visualized alongside antimicrobial resistance gene presence/absence data using iTOL v7.2.2 [27].

Moreover, five MDR *P. aeruginosa* isolates from this study were compared with 1032 *P. aeruginosa* genomes retrieved from the PubMLST database that belonged to the ST groups identified in this study [accessed on 7 November 2025]. In addition to filtering the dataset by ST groups, we applied an additional filter to include only isolates originating from countries neighboring Croatia—Bosnia and Herzegovina, Serbia, Montenegro, Slovenia, Slovakia, Italy and Hungary. Metadata, such as ST group, isolate origin, country, and epidemic status, were added as separate annotation columns. The resulting neighbor-joining trees and associated metadata were visualized using iTOL v7.2.2. Figures were finalized in Inkscape v1.3.2.

The genomic region surrounding the *bla*_VIM-2_ gene was extracted from the assembled contigs using BEDtools getfasta (v2.31.1) and annotated with CARD, Bakta, Prokka and mobile-OG implemented in the Proksee server [28]. Genomic context visualization was generated with Easyfig to compare and illustrate the genetic environment [29], while figures were finalized and formatted in Inkscape v1.3.2.

Potential horizontally acquired regions were identified using Alien_Hunter (v1.7.8). Contigs containing *bla*_VIM-2_ were analyzed, although two contigs were excluded from the analysis due to insufficient length (VAP-PA-1 and VAP-PA-3).

Putative plasmid-derived contigs were identified using MOB-suite (mob_recon module) with default parameters, which is a tool developed for plasmid identification and classification from short-read data. The putative plasmid-derived contig was compared against the NCBI plasmid database using BLASTn v.2.17.0 (megablast) to determine its similarity to known *P. aeruginosa* plasmid sequences. Class 1 integrons and associated cassette arrays were detected using IntegronFinder v2.0.

## 3. Results

A total of 49 non-repetitive *P. aeruginosa* isolates met the inclusion criteria and were tested for antimicrobial susceptibility during the study period.

### 3.1. Phenotypic Antimicrobial Susceptibility Testing (AST)

**Figure 1:**
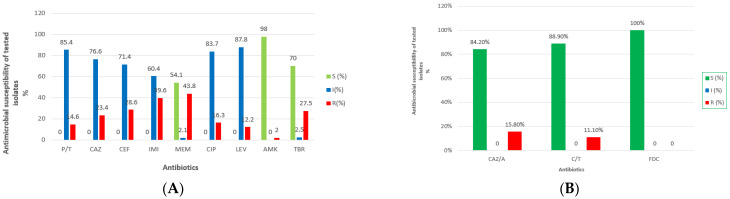
(**A**) Antimicrobial susceptibility pattern of 49 *P. aeruginosa* isolates; (**B**) Antimicrobial susceptibility of *P. aeruginosa* isolates tested to novel β-lactam/β-lactamase inhibitor combinations. Note: P/T, piperacillin/tazobactam; CAZ, ceftazidime; CEF, cefepime; IMI, imipenem; MEM, meropenem; CIP, ciprofloxacin; LEV, levofloxacin; AMK, amikacin; TBR, tobramycin; CAZ/A, ceftazidime/avibactam; C/T, ceftolozane/tazobactam; FDC, cefiderocol.

AST results of 49 isolates are shown in Figure 1A. High level of resistance was observed to IMI (39.6%), MEM (43.8%) and CEF (28.6%). An MDR phenotype was detected in 30.6% (15/49) isolates.

Among tested β-lactams, 48 isolates were evaluated for P/T, with 41 (85.4%) classified as susceptible with increased exposure, and 7 (14.6%) as resistant. Ceftazidime susceptibility was assessed in 47 isolates; 36 (76.6%) were categorized as susceptible with increased exposure, while 11 (23.4%) were resistant. All 49 isolates were tested for CEF, with 35 (71.4%) demonstrating intermediate susceptibility and 14 (28.6%) resistance. For IMI, 48 isolates were tested, of which 29 (60.4%) showed intermediate susceptibility and 19 (39.6%) were resistant. Meropenem susceptibility was detected in 26 isolates (54.1%), one isolate (2.1%) was intermediate, and 21 isolates (43.8%) were resistant.

All 49 isolates were tested for CIP, with 41 (83.7%) categorized as susceptible with increased exposure and 8 (16.3%) as resistant. Similarly, LEV intermediate susceptibility was observed in 43 isolates (87.8%), while 6 (12.2%) were resistant. Amikacin demonstrated the highest susceptibility among antibiotics, with 48 of 49 isolates (98%) classified as susceptible and only one isolate (2%) resistant. Of the 40 isolates tested for TBR, 28 (70%) were susceptible, one (2.5%) was intermediate, and 11 (27.5%) were resistant.

A subset of isolates (all MDR isolates and several randomly selected non-MDR isolates) underwent susceptibility testing against novel β-lactam/β-lactamase inhibitor combinations and results are shown in Figure 1B. Nineteen isolates were tested for CAZ/A susceptibility, with 16 (84.2%) susceptible and 3 (15.8%) resistant, while 18 isolates were tested for C/T, of which 16 (88.9%) were susceptible and 2 (11.1%) resistant. Results for ten MDR isolates tested for FDC were recorded and all isolates demonstrated full susceptibility (100%).

Additionally, colistin susceptibility results were available for nine MDR isolates, which were all susceptible, with minimum inhibitory concentrations (MICs) ranging from 0.5 to 4 mg/mL.

### 3.2. Whole-Genome Sequencing (WGS) Profiling and MLST Analysis

#### 3.2.1. Multilocus Sequence Typing (MLST) and Phylogenetic Analysis

Five MDR isolates (5/15; 33.3%) were randomly selected for WGS and additional molecular analysis was conducted. All selected *P. aeruginosa* (VAP-PA-1-5) isolates exhibited similar genome sizes (≈6.9–7.1 Mb) and GC content (~65.5%), while N50 values, numbers of coding sequences, and contig counts varied among the isolates (Table S1).

A MASH-based comparison revealed that each VAP-associated isolate showed the highest genetic similarity to a specific closely related reference genome. Specifically, VAP-PA-1 and VAP-PA-3 exhibited maximum similarity with GCF_027595025.1, whereas VAP-PA-2, VAP-PA-4, and VAP-PA-5 were most closely related to GCF_036233515.1, GCF_002085755.1, and GCF_045348545.1, respectively, with very low MASH distances indicating high genomic similarity (Figure S1A). Moreover, ANI-based clustering using fastANI showed the same overall pattern, confirming high nucleotide identity (>98.5% ANI) between each VAP isolate and its closest RefSeq counterparts (Figure S1B).

**Figure 2:**
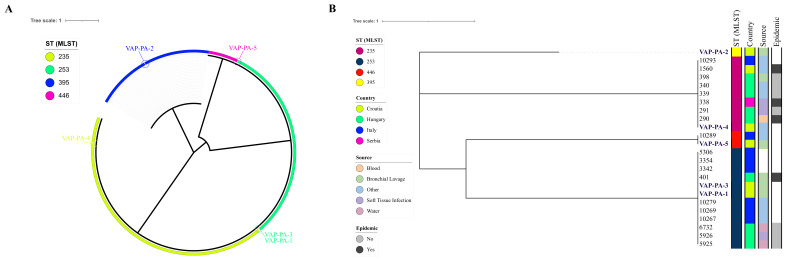
Phylogenetic analysis of five *P. aeruginosa* isolates. (**A**) Circular phylogenetic tree based on MLST allele profiles, including all PubMLST isolates belonging to the sequence type groups detected in this study (ST235, ST253, ST395, and ST446). VAP-PA isolates are highlighted and colored according to ST. (**B**) Hierarchical clustering dendrogram generated from a geographically filtered dataset including isolates originating from Croatia and neighbouring countries. Metadata panels indicate MLST, country of origin, source of isolation, and epidemic association. Tree scale bars represent nucleotide substitutions per site.

The isolates belonged to four different sequence types (STs): VAP-PA-1 and VAP-PA-3 were assigned to the high-risk ST253 and carried an identical set of AMR genes; VAP-PA-2 belonged to the widely distributed ST395; VAP-PA-4 to ST235; and VAP-PA-5 to ST446 (Figure 2).

The global phylogeny (Figure 2A) shows that the VAP-PA isolates clustered within other *P. aeruginosa* lineages of the mentioned ST groups. VAP-PA-1 and VAP-PA-3 grouped with high-risk global clones ST253 isolates, whereas VAP-PA-4 clustered within the widely disseminated high-risk clone ST235. Moreover, VAP-PA-5 clustered with other ST446 lineages associated with MDR profiles, while VAP-PA-2 was placed within another high-risk clone ST395 clade. To better understand the dissemination of these clones across Croatia’s neighbouring countries, we analyzed a subsample. The focused regional phylogeny (Figure 2B) further analysed these clusters and demonstrated clear separation into ST-specific groups. Isolates associated with known epidemic lineages were predominantly observed within the ST235 branch, consistent with its high-risk epidemiological profile. Moreover, only this ST group was found in all four countries from this analysis, originating mainly from hospital sources, not the environment. VAP-PA-1 and VAP-PA-3 remained closely related, suggesting potential local transmission or acquisition from a shared source. Clustering of isolates from this study with specimens from other countries supports the theory about high-risk MDR *P. aeruginosa* lineages being widely distributed.

#### 3.2.2. Antimicrobial Resistance Genomic Profile

**Figure 3:**
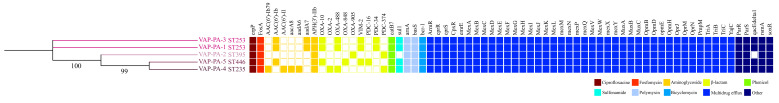
Phylogenetic tree and antimicrobial resistance profiles of five *P. aeruginosa* isolates. A maximum likelihood tree was constructed using core genome alignments. Branch lengths are scaled to the number of nucleotide substitutions per site, as indicated by the scale bar. Sequence types (STs) determined by MLST are indicated next to each isolate. The presence (colored) or absence (white) of antimicrobial resistance genes (ARGs), identified by ABRicate, is shown in the matrix on the right.

To facilitate comparison of genomic relatedness and AMR profiles among the *P. aeruginosa* isolates, phylogenetic relationships were analyzed alongside the distribution of resistance determinants (Figure 3). The best-fit evolutionary model (GTR + F + I) was selected based on the Bayesian Information Criterion (BIC) using ModelFinder and subsequently used to construct the phylogenetic tree. The resulting phylogenetic tree, displayed together with the AMR profiles of the five *P. aeruginosa* strains, shows both shared resistance determinants and strain-specific differences. Several intrinsic and acquired resistance determinants were conserved across all genomes, including genes associated with resistance to fluoroquinolones, fosfomycin, chloramphenicol, polymyxins, and bicyclomycin. Variability was mainly observed in genes associated with aminoglycoside and β-lactam resistance (Figure 3).

One isolate (VAP-PA-4, ST235) harbored a higher number of aminoglycoside resistance genes compared with the remaining strains. All isolates encoded chromosomally mediated β-lactam resistance mechanisms, including class C AmpC cephalosporinases and class D oxacillinases, whereas genes associated with acquired carbapenem resistance were detected only in a subset of isolates belonging to ST253 and ST446 (VAP-PA-1, VAP-PA-2 and VAP-PA-5).

In addition, genes encoding multiple efflux systems and their regulatory elements were consistently present across all genomes. Genes associated with resistance to sulfonamides and reduced susceptibility to antiseptics were detected in isolates belonging to ST253, ST446, and ST235, while the antiseptic resistance gene *qacEΔ1* was absent in VAP-PA-2.

#### 3.2.3. Virulence Genomic Profile

WGS analysis revealed the presence of numerous virulence genes, which are primarily part of the *P. aeruginosa* core genome and may contribute to its pathogenicity. An overview of virulence genes in all analyzed isolates can be found in Table S2. Isolates VAP-PA-1 and VAP-PA-3 have the same number of unique virulence-associated genes identified by VFDB (297 in total), which differs from other isolates (360 in VAP-PA-2, 290 in VAP-PA-4 and 303 in VAP-PA-5).

Of particular interest are genes responsible for adhesion, biofilm formation, quorum sensing and outer membrane proteins production (Table S2). Notably, all strains possess genes for exotoxin A and exoenzyme activity.

Distribution of the genes for the type 3 secretion system (T3SS) among the five isolates is as follows: VAP-PA-1, VAP-PA-3, VAP-PA-4 and VAP-PA-5 are *egzoY+egzoT+egzoU+egzoS*−, while VAP-PA-2 is *egzoY+egzoT+egzoU*−*egzoS+*.

However, as no functional assays or gene expression analyses were performed, the presence of these genes should be interpreted as genomic virulence potential, rather than confirmed phenotypic activity.

#### 3.2.4. Genetic Context of *bla*_VIM-2_ Isolates

**Figure 4:**
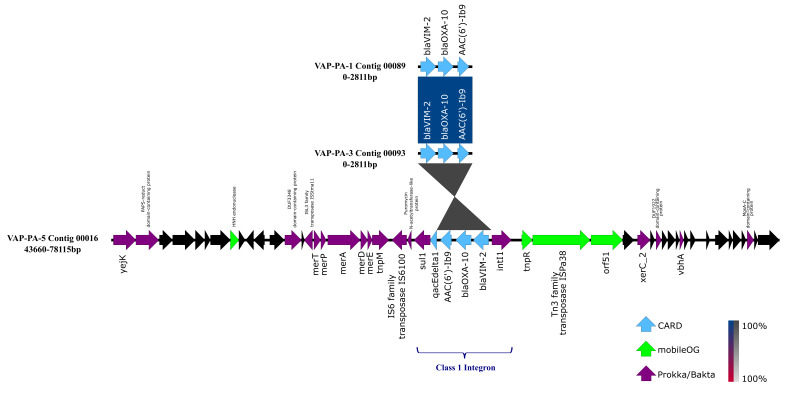
Comparison of the genetic environments of aminoglycoside genes (*aac(6′)-Ib9* and carbapenemase genes (*bla*_OXA-10_ and *bla*_VIM-2_) in three *P. aeruginosa* isolates (VAP-PA-1, VAP-PA-3 and VAP-PA-5). Shaded regions indicate nucleotide identity, with the red-blue indicating the same orientation, and light and dark grey indicating opposite orientation. Antimicrobial resistance genes are shown in blue arrows annotated by CARD, purple arrows represent CDS annotated by Prokka/Bakta, while mobile elements annotated by mobileOG were indicated with a green arrow. Black arrows indicate a hypothetical protein.

Three *P. aeruginosa* isolates (VAP-PA-1, VAP-PA-3 and VAP-PA-5) carried the *bla*_VIM-2_ metallo-β-lactamase gene and were selected for genetic context analysis (Figure 4). In VAP-PA-1 and VAP-PA-3, *bla*_VIM-2_ was located on a short contig that contained only three resistance genes—*aac(6′)-Ib9*, *bla*_OXA-10_ and *bla*_VIM-2_. The limited flanking sequence may reflect technical limitations related to assembly in regions rich in mobile genetic elements, but could also result from incomplete sequencing coverage or plasmid rearrangements. Moreover, the identical gene combination and order in those two and VAP-PA-5 suggest that all three isolates likely harbor the same cassette array embedded within a class 1 integron.

However, isolate VAP-PA-5 harbored the *bla*_VIM-2_ gene on a much larger contig (contig00016, 191,572 bp) carrying the full integron structure and surrounding genomic context. IntegronFinder identified a complete class 1 integron spanning positions 60,110–64,309 bp, comprising the canonical structure intI1—*aac(6′)-Ib9*—*bla*_OXA-10_—*bla*_VIM-2—_qacE∆1—*sul1*, with multiple attC sites and neighboring transposase genes (*tnpA*, *tnpR*) (Table S3). Upstream of the integron, a mercury-resistance operon (merA, merE, merP, merT) and several hypothetical proteins were detected.

MOB-recon classified contig00016 as putatively plasmid-derived (Table S4), and plasmid maintenance genes (parM, parB) were annotated by Prokka, supporting a plasmid-associated origin. BLAST comparison of contig00016 against GenBank plasmid sequences showed 100% identity and 90% query coverage to plasmid p3796A (Gen-Bank accession number: OX638565.1; 178 kb) from *P. aeruginosa* strain 3796A, indicating a high similarity to the p3796A plasmid backbone.

AlienHunter analysis of VAP-PA-5 identified five compositionally atypical genomic regions indicative of horizontal gene acquisition (Figure S2). The strongest anomaly (scores > 65) was located between 55–65 kb—directly overlapping the *aac(6′)-Ib9*—*bla*_OXA-10_– *bla*_VIM-2_ cluster—and thus strongly supporting the mobility and horizontal origin of this multidrug-resistance integron.

## 4. Discussion

LRTI caused by *P. aeruginosa* has been linked to longer ICU stays, extended durations of mechanical ventilation, complications of the underlying diseases and higher hospital costs. Therefore, this highly resistant and virulent bacterium continues to pose a significant global healthcare challenge with an urgent need for new therapeutics [30].

One of the mechanisms by which *P. aeruginosa* succeeds in causing severe, persistent LRTI is AMR. Resistance can arise through intrinsic mechanisms, as well as HGT, particularly affecting ß-lactams and aminoglycosides, that are frequently used in empirical and combination therapy [2]. In the present study, carbapenem resistance was frequently observed, while effective antipseudomonal cephalosporins were mostly susceptible with increased exposure. These findings are descriptive and should be interpreted cautiously, given the limited number of isolates analyzed. Moreover, *P. aeruginosa* has an outstanding ability to rapidly develop further resistance, thus making pseudomonal infections more severe and difficult to treat [31].

The incidence of treatment failure in patients with VAP caused by *P. aeruginosa* reaches up to 36%, even among those receiving combination antibiotic therapies [20]. A potential explanation is the high virulence potential of *P. aeruginosa*, particularly its ability to form biofilms, exotoxins, siderophores and immunomodulation [32,33,34]. The genomic potential of isolates from our study aligns with observations from other research [10]. However, many of these determinants are part of the *P. aeruginosa* core genome and their presence alone does not indicate increased virulence or predict disease severity. In the absence of functional assays, gene expression analyses or clinical outcome data, these findings should be interpreted strictly as genomic virulent potential.

Novel therapeutic approaches include alternative therapies whose action is directed at specific virulence factors. More precisely, phage therapy, quorum-sensing inhibitors, and immunotherapy are promising but still challenging options. Immunotherapy with recombinant anti-*Pseudomonas* PEGylated monoclonal antibody and anti-*Pseudomonas* immunoglobulin Y antibodies are in different clinical trial phases, while phage therapy is practiced in eastern European countries, like Poland and Georgia. Although there are no licensed vaccines, the study showed that an octavalent, polysaccharide, toxin-conjugate vaccine (Swiss Serum and Vaccine Institute) significantly lowered the rate of infection in cystic fibrosis patients [35]. However, many of these options are still hypothetical and need to be explored in future studies.

The results of susceptibility testing to novel combinations with beta-lactamase inhibitors (C/T and CAZ/A) and FDC have shown potential efficacy for the treatment of infections caused by MDR isolates. However, a limitation of these findings is the small sample size, so future studies are needed. Tazobactam and avibactam bind chromosomally encoded AmpC β-lactamase, while avibactam has anti-pseudomonal activity by itself (by unknown mechanism). However, the strong potential of efflux pumps and overproduction of AmpC beta-lactamases may result in clinical failure in the future [36,37].

The genetic environment of the *bla*_VIM-2_ gene in three *P. aeruginosa * isolates from this study demonstrates its association with an MDR locus, integrating multiple AMR determinants and mobile genetic elements. In isolate VAP-PA-5, the presence of a complete class 1 integron—containing *intI1*, the cassette array (*aac(6′)-Ib9*, *bla*_OXA-10_, *bla*_VIM-2_), and the 3′-CS genes qacE∆1–*sul1*—together with flanking transposases and a mercury-resistance operon, is consistent with integron structures commonly described in high-risk *P. aeruginosa* lineages [38,39,40]. AlienHunter analysis revealed that the *P. aeruginosa* VAP-PA-5 genome contains multiple horizontally acquired regions, with the most prominent segment (55–65 kb) corresponding to a locus enriched in resistance genes (*bla*_VIM-2_, *bla*_OXA-10_ and *aac(6′)-Ib9*). This clustering of β-lactamase and aminoglycoside resistance determinants within a compositionally atypical region is consistent with the well-documented association of such ARGs with class 1 integrons, transposons and MDR plasmids in *P. aeruginosa*, supporting their acquisition as part of a mobile MDR island [41].

BLAST analysis revealed high similarity to the plasmid p3796A, suggesting that the blaVIM-2-carrying integron is located within a putatively plasmid-derived-MDR region closely related to a known *P. aeruginosa* plasmid backbone found in an isolate from a urine sample of an individual in non-hospital settings in Bulgaria [42]. However, that study found that the plasmid lacked any resistance or virulence determinants, which is not the case in our study. In VAP-PA-5, the non-aligning region corresponds to an accessory module harboring a complete class 1 integron associated with transposase genes, IS6100 and a mercury-resistance (mer) operon. However, due to the use of short-read sequencing, the precise plasmid structure and genomic context of this region cannot be fully resolved; therefore, long-read sequencing technologies are required to reconstruct the plasmid architecture and confirm gene localization conclusively. Due to this limitation, any interpretations of plasmid structure or mobility should be treated cautiously. Nevertheless, these findings support the involvement of plasmid-associated horizontal gene transfer in the dissemination of antimicrobial resistance determinants among the studied isolates.

The detection of multiple additional AlienHunter-positive regions outside this AMR genes locus suggests that VAP-PA-5 may carry other horizontally transferred elements, potentially contributing to virulence, metabolic flexibility, or adaptation to hospital environments. Together, the structural, plasmid-homology, and compositional evidence strongly indicate that the *bla*_VIM-2_ integron represents a mobile, horizontally acquired resistance platform, capable of mediating multi-class antibiotic resistance and potentially facilitating dissemination across *Pseudomonas* populations.

The analyzed isolates belonged to four different sequence types (ST253, ST395, ST235 and ST446), all of which have been reported globally [10,43,44]. Phylogenomic analyses indicated that the isolates were genetically distinct and did not originate from a single clonal source, a finding further supported by MASH- and ANI-based clustering. While these sequence types have been associated with multidrug resistance in other studies [7], the small sample size precludes conclusions regarding regional dissemination, outbreak dynamics, or epidemiological trends.

Although on the dendrogram, VAP-PA-2 stays alone, the ST395 strain was found already in Hungary [44]. However, the absence of publicly available ST395 genome sequences in the database used for this study represents a limitation of the analysis and prevented its inclusion in the regional phylogenetic reconstruction. These findings emphasize the value of genomic-based surveillance to detect the emergence and spread of epidemic *P. aeruginosa* lineages in ventilator-associated pneumonia. Notably, the ST395 clone has widespread distribution in Europe, Asia and America and is the endemic, predominant clone in France [10]. Interestingly, it seems to have a lower virulence potential compared with a reference strain. More precisely, this clone has a 131 kb chromosomal deletion; therefore, genes involved in bacterial adherence, biofilm formation, and type IV pili are missing [10]. In agreement with the previous report, the ST395 isolate analyzed in this study lacked the *exoU* gene, which has previously been associated with more severe disease manifestations, and which was present in the remaining isolates [10].

A key limitation of this study is the small number of isolates subjected to WGS. Nevertheless, the data provide a detailed descriptive overview of resistance determinants, virulence-associated gene content and the genetic environment of *bla*_VIM-2_ in *P. aeruginosa* isolates associated with VAP. Future studies combining genomic, functional and clinical data will be essential to clarify the clinical relevance of these findings.

## 5. Conclusions

Understanding the molecular basis of resistance and virulence through WGS analysis is important, both locally, for the timely selection of effective therapy, and globally, for monitoring the epidemiological situation and evolution of high-risk clones.

## Data Availability

The data from this study have been deposited in the European Nucleotide Archive (ENA) under project PRJEB102320, and the accession numbers for each isolate are as follows: VAP-PA-1 (ERS27185630), VAP-PA-2 (ERS27185631), VAP-PA-3 (ERS27185632), VAP-PA-4 (ERS27185633) and VAP-PA-5 (ERS27185634).

## Supplementary Materials

The following supporting information can be downloaded at: https://www.mdpi.com/article/10.3390/genes17020130/s1, Figure S1: Comparative genomic similarity of VAP-associated *P. aeruginosa* isolates; Figure S2: AlienHunter prediction with ARG positions; Table S1: Metadata of the whole genome sequenced VAP isolates from Croatia; Table S2: Categories of virulence genes, their association to secretion system and corelated pathogenicity; Table S3: Class 1 Integron annotation—Integron Finder and ABRicate Results; Table S4: Mob Suite report for plasmid included in the study.

## Abbreviations

The following abbreviations are used in this manuscript:

VAP	Ventilator-associated pneumonia
VAT	Ventilator-associated tracheobronchitis
DTT	Difficult-to-treat
ICU	Intensive Care Unit
MALDI TOF MS	Matrix-Assisted Laser Desorption/Ionization Time of Flight Mass Spectrometry
AST	Antimicrobial susceptibility testing
AMR	Antimicrobial resistance
WGS	Whole-genome sequencing
MLST	Multi-locus sequence typing
VIM-2	Verona Integron-encoded Metallo-β-lactamase 2
HGT	Horizontal Gene Transfer
HAI	Hospital-acquired infections
PDC	Psedomonas-derrived cephalosporinase
MBL	Metallo-β-lactamase
WHO	World Health Organisation
MDR	Multi-drug resistant
LRTI	Lower respiratory tract infection
CFU	Colony-forming units
BA	Blood agar
P/T	Piperacillin/tazobactam
CAZ	Ceftazidime
CEF	Cefepime
CAZ/A	Ceftazidime/avibactam
C/T	Ceftolozane/tazobactam
FDC	Cefiderocol
CIP	Ciprofloxacin
LEV	Levofloxacin
IMI	Imipenem
MEM	Meropenem
AMK	Amikacin
TBR	Tobramycin
EUCAST	European Committee on Antimicrobial Susceptibility Testing
DNA	Deoxyribonucleic Acid
qPCR	Quantitative Polymerase Chain Reaction
N	Nucleotides
ARG	Antimicrobial Resistance Gene
MAFFT	Multiple Alignment using Fast Fourier Transform
ST	Sequence Type
NCBI	National Center for Biotechnology Information
BLAST	Basic Local Alignment Search Tool
MIC	Minimum inhibitory concentrations
APH	Aminoglycoside Phosphotransferase
AAC	Aminoglycoside acetyl-transferase
ANT	Aminoglycoside nucleotid-transferase
RND	Resistant-nodulation-division
QS	Quorum Sensing
