# Supplementary material for: Phenotypic and Whole-Genome Sequencing-Based Profiling of Antimicrobial Resistance and Virulence in Pseudomonas aeruginosa Isolated from Patients with Ventilator-Associated Pneumonia and Ventilator-Associated Tracheobronchitis in a Croatian Intensive Care Unit
Source: Genes (Basel). 2026 Jan 26;17(2):130. doi: 10.3390/genes17020130 (PMC12940210; doi:10.3390/genes17020130)
Supplement: Supplementary file 1 [file genes-17-00130-s001.zip › Suppl files, tables.pdf]

**Table S1. Metadata of the whole genome sequenced VAP isolates from Croatia**					
	**VAP-PA-1**	**VAP-PA-2**	**VAP-PA-3**	**VAP-PA-4**	**VAP-PA-5**
**Species**	*Pseudomonas aeruginosa*	*Pseudomonas aeruginosa*	*Pseudomonas aeruginosa*	*Pseudomonas aeruginosa*	*Pseudomonas aeruginosa*
**Total reads**	10,296,488	9,385,376	6,355,648	8,498,626	9,769,680
**Genome size (bp)**	7,052,379	6,975,685	7,052,091	6,920,520	7,054,791
**No. of CDS**	6489	6405	6488	6367	6552
**No. of contigs**	244	163	241	227	179
**No. of contigs > 1000 bp**	116	63	120	126	81
**Average depth (x)**	147,45	136,1	90,83	133,71	138,75
**GC content (%)**	65,58	65,61	65,51	65,31	65,45
**N50 (bp)**	188383	294341	144532	131139	196516
**tRNA**	64	70	64	68	66
**rRNA**	3	5	3	5	5
**tmRNA**	1	1	1	1	1

Table S2. Categories of virulence genes, their association to secretion system and correlated pathogenicity.

Pathogenicity and Virulence factors	Genes related to pathogenicity	Protein
		Type IV pili (T4SS)
	fimX, V	protein FimX, FimV
	xcpA/pilD	type 4 prepilin peptidase PilD
	pilB, T, U, G, H, I, J, K, Z, Q, P, O, N, M, R, S, F	twitching motility proteins, inner membrane proteins, two-component system
Adherence	*rpoN,S*	RNA polymerase sigma factors
	chpA, B, C, D, E	chemotaxis protein
	pscK, crc, vfr	regulatory proteins
		Fap
	fapA, B, C, D, E, F	minor and major amyloid subunits, chaperon and outer membrane secretin
		Flagellum (T6SS-associated)
Motility	*motA, B, Y, C, D,* PA3348, PA3349, PA1458, PA1459, PA1464*, fliK, L, M, N, O, P, Q, R, A, J, I, H, G, F, E, S, D, C flhB, A, F, N, fleN, R, S, Q; fleP/fliT fleI/flaG, cheY, Z, flgA, M, N, E, D, C, B, L, K, J, I, H, G, flgF*	multiple flagellar proteins, including flagellar motor protein, B-type flagellin, chemotaxis proteins, flagellar hook-associated protein, flagellar basal-body rod protein
		Alginate (exopolysaccharide)
Biofilm formation	*algW, A, F, J, I, L, X, G, E, K, 44, 8, D, B, U, C, Q, R, Z, algP/algR3*	outer membrane protein AlgE, alginate biosynthesis proteins, two-component response regulator AlgB regulatory proteins
	mucP, A, B, C, D, E	metalloprotease, small envelope protein MucE
		Quorum sensing (QS)

	lasR, lasI	transcriptional regulator LasR, autoinducer synthesis protein LasI
	rhlI, rhlR	transcriptional regulator RhlR, autoinducer synthesis protein RhlL
Multi-toxin components type 3 secretion system (T3SS) (cytotoxins injections, colonization, adhesion)	*exoU, exoT, exoS, exoY*	Exoenzyme (Exo): U, T, S, Y
	pscU, T, S, R, Q, P, O, N, *popN, B, D; pcr1, 2, 3, 4, pcrV, D, R, G, H exsC, E, B, A, D*	regulatory proteins
Multi-toxin components type 6 secretion system (T6SS)	*hcp1*<br>*vgrG1a, vgrG1b, clpV1, tse1, tse4, tse5/rhsP1, tse6*	Haemolysin coregulated protein/ Valine-glycinerepeat protein complex<br>AAA+ family ATPase<br>Toxin proteins Tse1 HIS-1, Tse4 HSI-1, Tse5 HSI-1, Tse6 HSI-1
Exotoxin A (ETA) (invasion, tissue lysis)	*toxA, plcH*	Exotoxin A, hemolytic phospholipase C
Immune modulation	*waaF, waaC, waaG, waaP, waaA, phzB2, exoY, rhlB, rhlA, rhlC* PA3142, PA3143	lipopolysaccharide core biosynthesis protein and enzymes, rhamnolipid
Lytic enzymes	*lasA, lasB*	elastase A, elastase B
	fimL, pldA/tle5a, pldB/tle5b, plcH	protease, phospholipase D, hemolysin phospholipase H
Siderophores		Pyoverdine
	PA2383, PA2384	transcriptional regulator
	pvdQ, A, P, M, N, O, F, E, Y, S, G, L, H	pyoverdine biosynthesis proteins
	fpvI, R, A	ferripyoverdine receptor FpvA
	ptxR, mbtH-like	MbtH-like protein from the pyoverdine cluste
	pvcA, B, C, D	paerucumarin biosynthesis protein
		Pyochelin
	fptA, PA4220, PA4219, PA4218	Fe(III)-pyochelin receptor precursor
	pchG, F, E, R, D, C, B, A, I	pyochelin biosynthetic proteins
Pyocyanin (toxic to host tissue)	*phzH, B1, A1, M, S, G2, F1, E1, D1, C1*	phenazine biosynthesis proteins

Table S3. Class 1 Integron annotation - Integron Finder and ABRicate Results													
**Integron Finder Results**													
# integron_finder 2.0.6													
ID_integron	ID_replicon	element	pos_beg	pos_end	strand	evalue	type_elt	annotation	model	type	default	distance_2attC	considered_topology
integron_01	contig00016	contig00016_86	60110	60457	-1	NA	protein	protein	NA	complete	No	NA	lin
integron_01	contig00016	attc_001	60560	60631	-1	1.3e-05	attC	attC	attc_4	complete	No	NA	lin
integron_01	contig00016	contig00016_87	60626	61216	-1	NA	protein	protein	NA	complete	No	NA	lin
integron_01	contig00016	attc_002	61325	61435	-1	2.9e-06	attC	attC	attc_4	complete	No	694.0	lin
integron_01	contig00016	contig00016_88	61430	62230	-1	NA	protein	protein	NA	complete	No	NA	lin
integron_01	contig00016	attc_003	62245	62317	-1	0.012	attC	attC	attc_4	complete	No	810.0	lin
integron_01	contig00016	contig00016_89	62329	63129	-1	NA	protein	protein	NA	complete	No	NA	lin
integron_01	contig00016	contig00016_90	63296	64309	1	2,20E-08	protein	intI	intersection_tyr_intI	complete	No	NA	lin

**Abricate Results**													
SEQUENCE	START	END	STRAND	GENE	COVERAGE	COVERAGE_MAP	GAPS	%COVERAGE	%IDENTITY	DATABASE	ACCESSION		
contig 00016	59277	60116	-	sul1	1-840/840	===============	0/0	100	100	**card**	JF969163.1:1053-1893		
contig 00016	60110	60457	-	qacEdelta1	1-348/348	===============	0/0	100	100	**card**	U49101.1:1490-1838		
contig 00016	60626	61216	-	AAC(6')-Ib9	25-612/612	===============	0/0	96,08	99,83	**card**	AF043381.1:251-863		
contig 00016	61430	62230	-	OXA-10	1-801/801	===============	0/0	100	100	**card**	AF205943.1:7511-8312		
contig 00016	62329	63129	-	VIM-2	1-801/801	===============	0/0	100	100	**card**	EF614235.1:2948-3749		

**Table S4. Mob Suite report for plasmid included in the study**																							
**sample_id**	**Info**	**num_contigs**	**size**	**gc**	**md5**	**rep_type(s)**	**rep_type_access**	**relaxase_type(**	**relaxase_type_accessio**	**mpf_type**	**mpf_type_accession**	**orit_type(s)**	**orit_accession(**	**predicted_mobil**	**mash_nearest_neigh**	**mash_neighbor_dista**	**mash_neighbor_identification**	**primary_cluster_**	**secondary_cluste**	**predicted_host_range_overall_r**	**predicted_host_range_overall_name**	**observed_host_range_ncbi_rank**	**observed_host_range_ncbi_na**

							**ion(s)**	**s)**	**n(s)**		**(s)**		**s)**	**ity**	**bor**	**nce**		**id**	**r_id**	**ank**			**me**
VAP-PA-5C	Strain from this study	1	191572	0.5945284279539808	f9f4c95041482af5b4adf7d2e572b2b5	-	-	-	-	-	-	-	-	non-mobilizable	CP041355	0.0184352	Pseudomonas aeruginosa	AC882	AM239	genus	Pseudomonas	genus	Pseudomonas
