# Supplementary figures and images for: Phenotypic and Whole-Genome Sequencing-Based Profiling of Antimicrobial Resistance and Virulence in Pseudomonas aeruginosa Isolated from Patients with Ventilator-Associated Pneumonia and Ventilator-Associated Tracheobronchitis in a Croatian Intensive Care Unit
Source: Genes (Basel). 2026 Jan 26;17(2):130. doi: 10.3390/genes17020130 (PMC12940210; doi:10.3390/genes17020130)

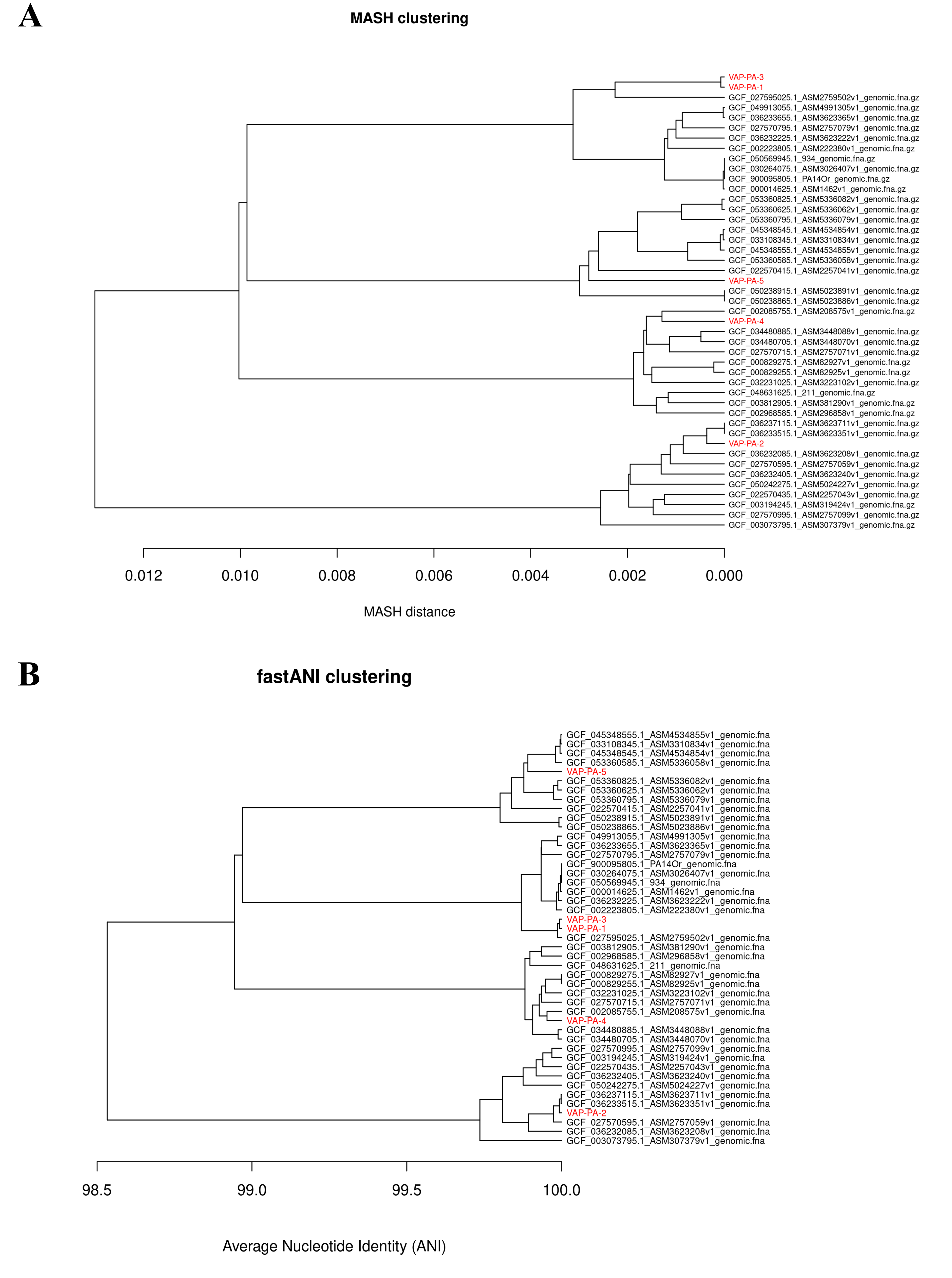

Supplement: Supplementary file 1 [file genes-17-00130-s001.zip › Figure S1 FinalMASHANI.png]

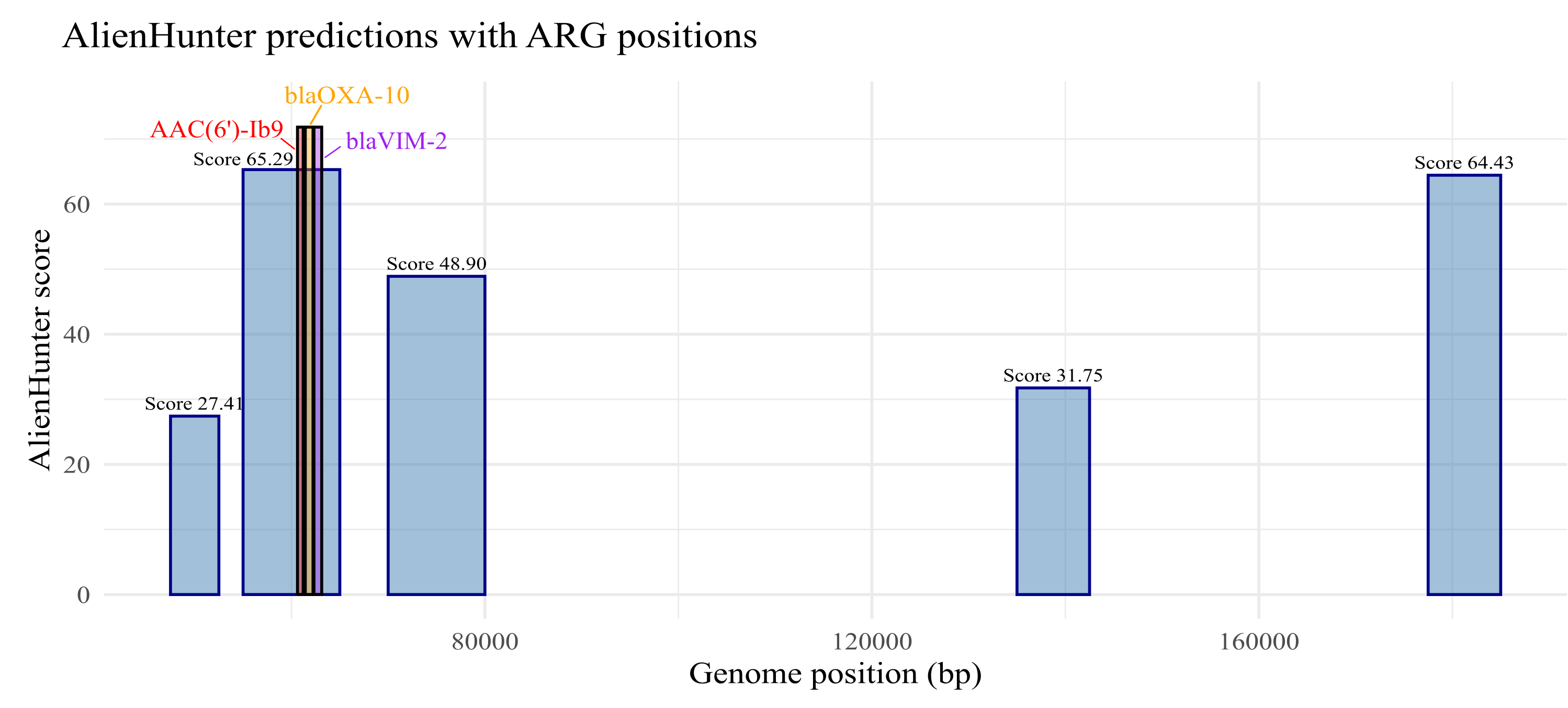

Supplement: Supplementary file 1 [file genes-17-00130-s001.zip › Figure S2 AlienHunterV5kontigF.png]
